# Deconvoluting human Brodmann area 8 based on its unique structural and functional connectivity

**DOI:** 10.3389/fnana.2023.1127143

**Published:** 2023-06-22

**Authors:** Nicholas B. Dadario, Onur Tanglay, Michael E. Sughrue

**Affiliations:** ^1^Robert Wood Johnson Medical School, Rutgers University, New Brunswick, NJ, United States; ^2^Omniscient Neurotechnology, Sydney, NSW, Australia

**Keywords:** Brodmann area 8, network, connectivity, cognition, fMRI, neuroimaging

## Abstract

Brodmann area 8 (BA8) is traditionally defined as the prefrontal region of the human cerebrum just anterior to the premotor cortices and enveloping most of the superior frontal gyrus. Early studies have suggested the frontal eye fields are situated at its most caudal aspect, causing many to consider BA8 as primarily an ocular center which controls contralateral gaze and attention. However, years of refinement in cytoarchitectural studies have challenged this traditional anatomical definition, providing a refined definition of its boundaries with neighboring cortical areas and the presence of meaningful subdivisions. Furthermore, functional imaging studies have suggested its involvement in a diverse number of higher-order functions, such as motor, cognition, and language. Thus, our traditional working definition of BA8 has likely been insufficient to truly understand the complex structural and functional significance of this area. Recently, large-scale multi-modal neuroimaging approaches have allowed for improved mapping of the neural connectivity of the human brain. Insight into the structural and functional connectivity of the brain connectome, comprised of large-scale brain networks, has allowed for greater understanding of complex neurological functioning and pathophysiological diseases states. Simultaneously, the structural and functional connectivity of BA8 has recently been highlighted in various neuroimaging studies and detailed anatomic dissections. However, while Brodmann’s nomenclature is still widely used today, such as for clinical discussions and the communication of research findings, the importance of the underlying connectivity of BA8 requires further review.

## 1. Introduction

**FIGURE 1:**
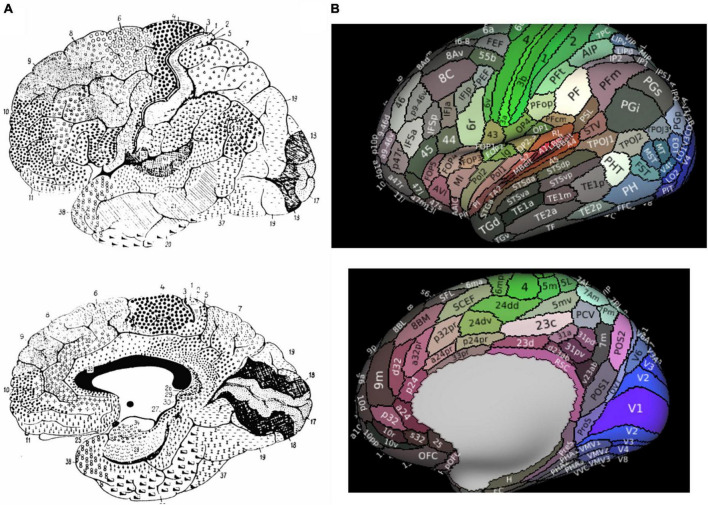
Parcellated Human Cerebrum. Panel **(A)** presents Brodmann’s original atlas. Panel **(B)** presents the 180 cortical parcellations described by the Human Connectome Project (HCP). The color of each parcellation is based on a 3D color space, reflecting the extent to which each areas is associated in the resting state with auditory (red), somatosensory (green), visual (blue), task positive (white), or task negative (black) groups of areas.

The human cerebral cortex has been divided into several different cortical maps over previous decades through a variety of analytical methods. Starting in the early 20th century, the human cerebrum was mostly divided by characterizing histological differences between regions according to their function. Brodmann’s map, the most widely used traditional map of the human brain, characterized the cerebral cortex into 43 regions according to regional cytoarchitectural differences in cells and laminar structures (Amunts and Zilles, 2015; Figure 1A). Brodmann originally defined BA8 as the posterior aspect of the superior frontal gyrus (SFG), extending medially to the paracingulate sulcus, posteriorly bound by area 6 and anteriorly by areas 9 and 46 (Petrides and Pandya, 2012). Specifically, Brodmann’s definition included the following: “Area 8–the intermediate frontal area–consists of a strip-like zone, wide superiorly and narrowing laterally, which, like the agranular frontal area (6), crosses from the callosomarginal sulcus on the medial surface over the upper edge of the hemisphere onto the lateral surface; but there it only reaches to about the middle frontal gyrus before gradually vanishing without distinct borders. Especially on the lateral convexity of the hemisphere it is much less extensive than area 6” (Garey, 1994). Several other cyto- (von Economo and Koskinas, 1925; Bailey, 1951; Sarkissov et al., 1955; Petrides and Pandya, 2012) and myleoarchitectural (Vogt and Vogt, 1919) studies have further divided BA8 into numerous subdivisions. In these studies, BA8 has been separated into area 8A on the middle frontal gyrus (MFG) (commonly said to be the “FEF”) (Lanzilotto et al., 2013), later with ventral (area 8Av) and dorsal (area 8Ad) components, as well as area 8B on the superior frontal gyrus (SFG) extending to the paracingulate sulcus (Petrides and Pandya, 2012). Importantly, despite utilizing similar methodology of anatomical delineations, all of the above maps differ significantly in their configuration, size, and number of cortical regions (Zilles and Amunts, 2010). Reasons for the limitations in these purely anatomical schemes have been discussed previously (Zilles and Amunts, 2010), but in general they are largely hindered by their single unit of neurobiological property, mostly cytoarchitectonic, combined with limited sample sizes which increase inter-subject variability.

Advances in neuroimaging capabilities and techniques for structural and functional imaging have led to an improved characterization of Brodmann’s maps. Of particular importance has been that of the Human Connectome Project (HCP) given their creation of a multi-modal atlas based on a comprehensive method combining architectural, functional, neural connectivity, and topographical differences between cortical regions in healthy individual brains. The HCP atlas identified a total of 180 fine cortical parcellations per cerebral hemispheres according to these various neurobiological properties (Figure 1B).

According to the HCP, the dorsolateral prefrontal cortex contains 4 subdivisions of BA8 (8BL, 8Ad, 8Av, and 8C) and two transitional areas between areas 6 and 8 were also described by the HCP (s6-8 and i6-8), while area 8BM is in the medial prefrontal cortex (Figure 1B; Glasser et al., 2016). What becomes particularly important with the new HCP scheme is how they redefined what is generally considered the pre-supplementary motor area (SMA) according to Brodmann, which has been subject to debate by others as well (Ruan et al., 2018). Generally, the dorsal medial frontal cortex contains both the SMA and pre-SMA (Ruan et al., 2018). According to Brodmann, the pre-SMA was included in area 8. However, the HCP authors separated area 8 from the pre-SMA, now anatomically designating the supplementary and cingulate eye field (SCEF) and superior frontal language (SFL) area as the pre-SMA, although they generally refer to these two regions along with areas 6ma and 6mp as the SMA in total (Glasser et al., 2016; Sheets et al., 2021).

## 2. The new anatomy of BA8–the basic anatomical and structural-functional connectivity patterns

The work by the HCP authors has undoubtedly provided us a significant body of information about structural and functional relationships of the human brain according to a more anatomically specific parcellated atlas. To build off of this work which predominantly explained the atlas using unfamiliar and non-anatomic based maps (e.g., flat maps which do not explain gyri and sulci in depth), we have previously described all 180 HCP parcellations in each hemisphere according to the surrounding cortical anatomy, functional connectivity, and structural connectivity (Baker et al., 2018b).

**FIGURE 2:**
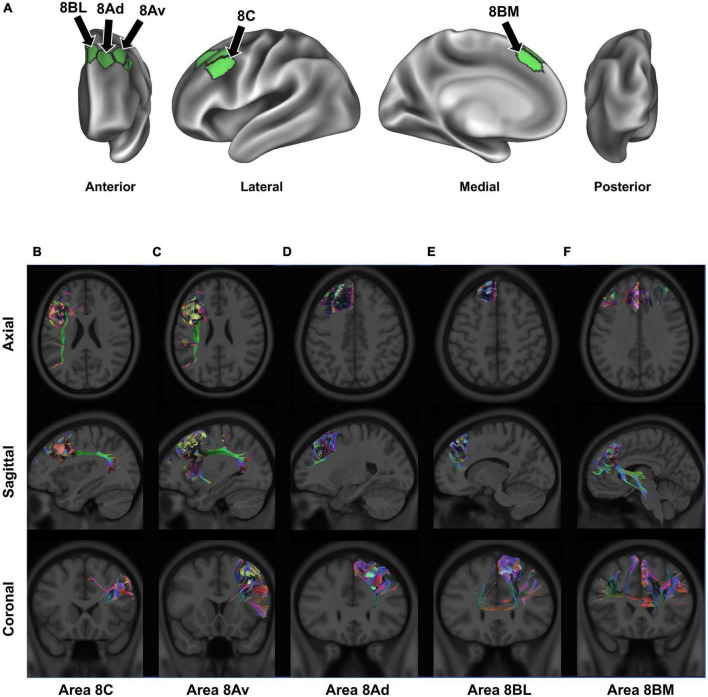
Connectivity of BA8. Panel **(A)** details the anatomical location of each BA8 subdivision, projected onto a left-hemispheric model brain. Panels **(B–F)** display the structural connectivity of all 5 area 8 subdivisions through tractography, projected onto a sample MRI image: area 8C **(B)**, area 8Av **(C)**, area 8Ad **(D)**, area 8BL **(E)**, and area 8BM **(F)**. Note that there may be parallax error within the projections, given the two-dimensional nature of the images.

In our definition, and in accordance with work by the HCP, BA8 can be divided into five regions: areas 8BL and 8AD on the posterior half of the superior frontal gyrus, areas 8AV and 8C on the posterior half of the middle frontal gyrus, and area 8BM in the medial superior frontal gyrus (Figure 2A; Baker et al., 2018a,c). Furthermore, two hybrid areas between areas 6 and 8 were also described by the HCP (s6-8 and i6-8) as well as pre-SMA areas SCEF and SFL but are not described in detail in the current work [see Glasser et al. (2016)]. We describe these regions further below in the context of their structural connectivity and speculated functional relevance (Figures 2B–F) (Glasser et al., 2016). For additional definitions and reasons for separating these subdivisions from other surrounding areas see the Supplementary material of Glasser et al. (2016) (specifically, Supplementary Figure 25; Glasser et al., 2016).

### 2.1. Areas 8BL and 8AD

Areas 8BL and 8AD can be found in the superior frontal gyrus. Area 8BL is located at the posterior aspect of the superior SFG surface. It is a lateral division of BA8, bounded medially by area 8BM, anteriorly by areas 9p and 9m, and posteriorly by areas s6-8 and the superior frontal language (SFL) area (Figure 1B). Area 8BL demonstrates a wide degree of functional connectivity throughout the frontal lobe, especially to other BA8 subdivisions in the dorsolateral prefrontal cortex, the middle and inferior frontal cortices, as well as the temporal lobe (e.g., temporal area 1 and 2 and the superior temporal sulcus areas) and the parietal lobe (e.g., areas 7 m and divisions of areas 31 and 23). Importantly, area 8B in macaques is commonly believed to be the premotor eye-ear field, and given the role of the posterior aspect of the SFG in working memory, area 8BL has been implicated in spatial working memory (Courtney et al., 1998). We have found that the major fiber bundle connecting area 8BL is also involved in higher visual-cognitive processes, specifically the inferior fronto-occipital fasciculus (IFOF) (Conner et al., 2018). Numerous divisions of the IFOF have been provided, and 8BL may be specifically connected via the IFOF-V which connects with numerous aspects of the occipital and parietal lobes (Wu et al., 2016). Previous work using DTI-tractography have found these connections travel from 8BL through the extreme/external capsule ending at occipital parcellations V2, V3, 7PL, MIP, V6, and V6A (Conner et al., 2018). Another major fiber bundle connecting 8BL are contralateral connections through the genu of the corpus callosum to end at contralateral 8BM and 9m, connections to the medial thalamus via the internal capsule, and frontal aslant tract (FAT) connections to the inferior frontal gyrus to terminate at area 44.

Compared to more medially located area 8BL, area 8Ad is located on the bank of the superior frontal sulcus as it joins the union between the SFS and precentral sulci. It is bordered anterior by areas 9p, 9-46d, and 46, laterally by area 8AV, and posteriorly by the transition areas s6-8 and i6-8. Similar to area 8BL, area 8Ad demonstrates extensive functional connectivity throughout the dorsolateral frontal cortices with area 8 and 10 subdivisions, MFG areas 24 and 32, and numerous temporal and parietal areas (e.g., subdivisions of area 7, 31, 23, and the hippocampal and parahippocampal gyri). However, unlike area 8BL, this region is more locally connected and highly inconsistent between individuals. We discuss the importance of these local connections in the next section, but they reflect the hub like nature of area 8Ad in the SFS, which may integrate visual and auditory information for spatial cognition via short local association bundles with areas 9a, 9p, s6-8, 8Av, and p10p (Reser et al., 2013).

### 2.2. Areas 8AV and 8C

Areas 8AV and 8C can be found on the middle frontal gyrus, with area 8AV on its most posterior aspect bound laterally by area 8C. Furthermore, area 8AV is bound anteriorly by area 46, posteriorly by areas 55b, FEF, and i6-8, and medially by area 8D. Interestingly, area 8AV demonstrates a number of similar functional connections as seen above with area 8BL, which we later describe as likely being related to their similar functional network associations. However, area 8AV is structurally connected primarily via the arcuate/superior longitudinal fasciculus (SLF), contralateral connections through the body of the corpus callosum to the contralateral superior frontal language area, and local association fibers. Arcuate/SLF fibers can be seen structurally connecting area 8AV to the parietal lobe after wrapping around the sylvian fissure posteriorly, while local association fibers connect it within BA8 with subdivisions area 8C, 8Ad, i6-8, and 46.

Area 8C is also located in the posterior aspect of the MFG, but bordered laterally by inferior frontal sulcus areas (IFSp, IFJa, and IFJp), posteriorly by the precentral eye field and area 55b, and anteriorly areas p9-46v and 46. Similar to medial area 8AV, area 8C can bee seen demonstrating functional connectivity with some similar frontal, temporal, and parietal regions, although some differences become apparent. Namely, area 8C demonstrates less functional connectivity with subdivisions of area 9 and more connectivity with inferior frontal lobe regions (IFSp, IFJp, a47r, p47r, and 44). However, a number of similar structural connections are also found between the two regions as area 8C is connected via the arcuate/SLF as well, but instead terminates in parietal visual areas PH and PHT unlike how the connections of 8AV via the arcuate/SLF terminate in different parietal areas (6a, 7PC, MIP, PFm, 2), which are largely implicated in praxis (Shahab et al., 2022).

### 2.3. Area 8BM

Area 8BM can be found on the posterior aspect of the medial SFG. Its superior boundary includes subdivision area 8BL and the SFL and area 24 subdivisions, areas d32 and a32pr inferiorly, area 9m anteriorly, and the supplementary and cingulate eye field (SCEF) posteriorly. Area 8BM has a particularly interesting amount of cross-modal functional connectivity as it can be seen linking a variety of different brain regions involved in different brain networks. In particular, area 8BM demonstrates functional connectivity with all area 8 subdivisions as well as areas i6-8, s6-8, a10p, a9-46v, and p9-46 in the dorsolateral frontal lobe, temporal regions TE1p, TE1m, and STSvp, as well as significant functional connectivity with numerous lateral parietal (e.g., LIPv, IP, and PG areas) and medial parietal (e.g., 7pm, 31a, and d23ab) regions. Unsurprisingly, this region is connected to numerous regions by both large fiber bundles and short local association fibers. Large fiber bundles via the IFOF connect area 8BM through the temporal lobe to end at parietal area 7PC and occipital areas V1-3, while FAT fibers connect area 8BM infero-laterally to area 44. Thalamic connections to the brainstem and contralateral connections to area 8BM and 9 m are also appreciated.

## 3. Connectivity of BA8 subregions determine their behavioral correlates

As a result of the cytoarchitectural boundaries of BA8 and its subdivisions being similar, it is reasonable to consider BA8 and its subdivisions facilitate the same functions. Ultimately, BA8 can generally be considered as a decision maker which is important in weighing uncertainty (Volz et al., 2004, 2005). However, what is important to consider is that the contexts differ in their activations based on who else they are structurally and functionally connected to. According to the literature, beyond traditional views suggesting BA8 is primarily a frontal eye field involved region, its association with a variety of higher-cognitive functions has been recently well-appreciated and well-documented. Neuroimaging based studies have implicated this region in motor learning (Matsumura et al., 2004) and imagery (Malouin et al., 2003), executive functions (Kübler et al., 2006), language (Fox et al., 2000; De Carli et al., 2007), working memory (Rämä et al., 2001), visuospatial attention (Cheng et al., 1995), and a number of other functions.

One major advancement in thinking provided by recent large-scale neuroimaging technology which can address this complex phenomenon is the understanding that higher-order cognitive functions cannot often be reliably linked to single cortical regions, and instead may be better understood based on the underlying connectivity of a region with different areas. From a network perspective, spatially distinct regions are functionally connected within large-scale brain networks to subserve complex human functions. Furthermore, functionally connected regions are commonly structurally connected by white matter connections, which place important constraints on functional connectivity and overall information processing (Bressler and Menon, 2010). This connectomic framework allows us to better understand BA8 and its subdivisions as likely an important hub in mediating different dynamic intra- and inter-network interactions between various large-scale brain networks to facilitate uncertainty driven decision making for processes determined by regions they are connected to. In other words, regardless of the reason for uncertainty (i.e., external or internal stimuli), activation in BA8 increases with increasing uncertainty, but the different ways to resolve or cope with this strategy is facilitated by which additional networks are activated (Volz et al., 2005). We expand on these principles below with common examples provided by recent literature.

### 3.1. Flexible decision making and memory

The prefrontal cortex has long been implicated in goal-directed behavior (Botvinick and An, 2009; Yang et al., 2022). In particular, the role of BA8 as a decision maker, such as for goal-directed behaviors, can likely be first appreciated by understanding the role of this region in working memory (WM). Important in guiding goal-directed behaviors includes the process of WM, which relies on the quick storage and manipulation of relevant information to guide subsequent behavior. Lesion based and electrophysiological studies including both humans and non-human primates have generally implicated the lateral prefrontal cortex as a predominant area facilitating these processes (Goldman-Rakic, 1987; du Boisgueheneuc et al., 2006; Luria, 2012; Fuster, 2015). WM tasks highlight the activation of SFG, and similarly damage to the SFG causes an impairment in working memory, especially spatially related WM (du Boisgueheneuc et al., 2006). This anatomic region generally corresponds to areas 8AD and 8BL. However, other subdivisions of BA8 have also been implicated by these processes, such as the 2-back test for area 8C and spatial relations for areas 8Av and 8C [see Supplementary Figure 25 in Glasser et al. (2016)]. Importantly, it is likely that BA8 does not facilitate working memory in a single domain (e.g., only visual or spatial), but rather these processes vary according to their specific connections. When examining Figure 1B by the HCP atlas, one can see that BA8 subdivisions differ in their functional activation across various cognitive domains. Others have referred to this process as “executive processing” (Postle et al., 2000), where for instance the SFG activates not only for processing of spatially related information, but rather represents a more flexible system for general cognitive control (Duncan and Owen, 2000; du Boisgueheneuc et al., 2006).

**FIGURE 3:**
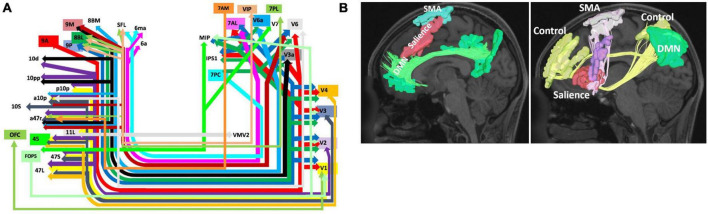
Network Interactions of BA8. Higher-order cognitive processes like goal directed behavior and motor planning and initiation are likely supported by the connectivity of BA8 to the visual system via the inferior fronto-occipital fasciculus (IFOF) **(A)** (Conner et al., 2018) and the interaction of BA8 with the salience, default mode, and control networks comprising an initiation axis spanning the middle frontal lobe (Poologaindran et al., 2020) **(B)**. Note that schematic in panel **(A)** shows the entire start and end points of the IFOF, which include areas 8BM and 8BL. Panel A was reproduced with permission from Conner et al. (2018) and Panel **(B)** with permission from Poologaindran et al. (2020).

One aspect of the connectivity of this region which may explain this functional relevance is the connectivity of BA8 via the IFOF system (Figure 3A). The IFOF bundle is a major white matter connection likely to be involved in higher cognitive processing through multiple connectivity related links with many networks. In particular, areas 8BL and 8BM have numerous connections throughout the cerebrum which may be facilitated via this system. As seen in Figure 3A, 8BL is primarily connected to earlier visual areas (V2-V4) and also the superior parietal lobe (e.g., 7PC and MIP), while area 8BM primarily sends information to later visual areas. Given the network affiliation of 8BL in the default mode network (DMN), it is possible these connections are likely determining cognitively relevant representations of the visual system (Buckner, 2013), which may subsequently facilitate functions such as praxis (O’Neal et al., 2021). Differently, as we discuss further in the next section, area 8BM is a central executive network (CEN) region which is anatomically located between two SMA regions. Area 8BM may likely facilitate the motor planning and execution of goal-directed behaviors through interacting with numerous higher order networks and the motor system along the medial frontal lobe (Mandonnet et al., 2017; Briggs et al., 2021a). Furthermore, BA8 has been implicated in various language functions, such as speech motor programming (Fox et al., 2000), language processing (De Carli et al., 2007), and translation (Price et al., 1999). Unsurprisingly, language areas such as area 44 show up on the IFOF system, and are connected to BA8 subdivisions like areas 8BL and 8C.

Ultimately, the role of BA8 as a decision maker and in working memory facilitates a number of functions according to this regions connectivity throughout the cerebrum and with the visual system. In particular, the IFOF is a major white matter bundle involved in higher-order cognitive processes beyond basic visual processing, and this system is likely one source of structural connectivity for BA8 which economically supports and constrains these functions. Importantly however, much of the results supporting these connectivity relationships between IFOF and medial area 8 regions (8BL, 8BM) has been provided through neuroimaging based work, such as using DSI tractography (Wu et al., 2016; Conner et al., 2018). With the increase in neuroimaging based techniques to map various aspects of the brain connectome, it is critical that these relationships are also verified with direct anatomic dissection as well, such as post-mortem dissections (Martino et al., 2010; Briggs et al., 2021b). Such direct evidence is lacking with the IFOF and medial BA8 regions to date, and therefore is an important area of future work to better understand the importance of this connectivity or lack thereof.

### 3.2. Decision making for motor control

A number of studies have implicated BA8 in goal-directed behavior, particularly for motor actions and conflict processing (Usami et al., 2013; Ben Shalom and Bonneh, 2019). A large reason for this focus of study likely originates from the fact that part of the traditional definition of BA8 according to Brodmann includes the pre-supplementary motor area (pre-SMA). However, the pre-SMA was later separated out by the HCP to predominantly include areas SCEF and SFL as discussed previously. Despite these differences in nomenclature, involvement of BA8 in motor planning and actions can be understood based on its underlying neural connectivity. One particular BA8 subdivision, area 8BM, is a CEN region which is strategically placed between these two DMN regions (SCEF and SFL). 8BM has numerous local structural connections with these two regions. This becomes particularly important as multiple lines of evidence have suggested a likely connectomic initiation axis responsible for facilitating motor planning spanning the medial frontal lobe (Figure 3B; Darby et al., 2018; Poologaindran et al., 2020; Briggs et al., 2021a). While 8BM is not known to be a direct part of the initiation axis, it likely interacts with other regions within the axis. This initiation axis consists of the DMN linked by the cingulum bundle and the salience network linked by the FAT, and it extends up to the SMA. Damage to the axis causes akinetic mutism and abulia, while sparing the axis prevents these deficits (Briggs et al., 2021a). Given area 8BM’s position between both DMN affiliated motor planning areas SCEF and SFL, as well as its major connections via the FAT, area 8BM’s role in overall motor planning and the initiation of goal-directed behavior is not entirely surprising. Furthermore, like area 8BM, SMA regions also are connected through the IFOF system further suggesting the importance of these connections in motor planning and execution.

## 4. Impaired BA8 connectivity and potential therapies

Given the role of BA8 in uncertainty driven decision making, it is important to consider how a lack of this neural correlate, such as in disease or following a lesion, has a notable amount of likely clinical importance. Generally, dysfunction in this region has been implicated in a variety of psychiatric illnesses [i.e., depression (Rogers et al., 2004; Siegle et al., 2007; Holmes and Pizzagalli, 2008) and obsessive-compulsive disorder (OCD) (Rotge et al., 2010; Yun et al., 2017)], behavioral disorders [i.e., ADHD (Hai et al., 2022)], neurodegenerative disorders [i.e., dementia (Godefroy et al., 2022) and Parkinson’s disease (Shen et al., 2020)], as well as motor (Bannur and Rajshekhar, 2000; Dadario et al., 2021) and language (Rubens et al., 1976; Freedman et al., 1984; Rapcsak and Rubens, 1994) deficits. Together, these deficits can be thought of as a lack of motivation, apathy, and poor response inhibition (Hu et al., 2016). However, what is important to note is that just considering BA8, or even perhaps its subdivisions, as prominent features in all of these disorders does not create an adequate model to actually better understand, treat, and prevent these symptoms. As an example, preventing damage to the SFG does not always prevent SMA syndrome, characterized by transient hemiparesis and akinetic mutism and abulia, and damage outside the SFG can still cause SMA syndrome (Ruan et al., 2018). Furthermore, not all patients recover and trajectories are unpredictable (Abel et al., 2015). However, as mentioned above, by considering the dynamic underlying structural and functional connectivity of this region and with other brain networks, we may be able to better understand these clinical diseases and also prevent them.

In resective brain surgery around BA8, connectivity features provide a map which may be utilized intraoperatively to avoid critical networks, such as by the SFG bank (Briggs et al., 2021a). Elsewhere, this connectomic architecture may also allow us to better understand heterogenous clinical symptomology associated with various neuropsychiatric illness related to this region. fMRI analyses have suggested network-based executive dysfunction in OCD is associated with different resting-state connectivity disturbances between an anterior cingulate component of the salience network and (1) the left dorsolateral BA8 (presumably 8C/8AV) for information integration and overall planning and (2) the superior lateral BA8 (presumably 8Ad/8BL) bilaterally for selective attention and response inhibition (Yun et al., 2017). Differently, dysfunctional connectivity in depression is demonstrated between SFG components of BA8 and default mode network nodes in the precuneus (Helm et al., 2018; Tanglay et al., 2022). Importantly in this context, various neuromodulatory treatments targeted in this region are now available to treat psychiatric disorders (Marques et al., 2019) and modulate specific behaviors (Rose et al., 2011), presumably by influencing surrounding the neural connectivity and (re)-synchronizing brain networks. Thus, simultaneously improving our understanding of the specific neural connectivity in this region can provide more precise information to identify anatomically specific targets for neuromodulatory treatments which are now capable of utilizing this level of granular information (Stephens et al., 2021; Einstein et al., 2022; Poologaindran et al., 2022).

## 5. Conclusion

A significant amount of information has been revealed about the anatomy of BA8 which has both challenged the traditional anatomic boundaries of this region and also expanded our understanding of its functional relevance. BA8 and its subdivisions are generally implicated in uncertainty driven decision making. However, this region is implicated in a variety of higher-order cognitive processes as the context of the decision making, and therefore activation of BA8, depends on its structural and functional connectivity to other brain regions and throughout various large-scale brain networks. These processes are largely evident through underlying multi-network interactions stemming from BA8, especially with the DMN and CEN, and communication through major fiber bundles like the (1) IFOF with the visual system and (2) connectomic initiation axis for goal-directed behavior and motor initiation.

## Author contributions

ND, OT, and MS: conception, writing, editing, reviewing, and figures. All authors contributed to the article and approved the submitted version.

## References

[B1] Abel T. Buckley R. Morton R. Gabikian P. Silbergeld D. (2015). Recurrent supplementary motor area syndrome following repeat brain tumor resection involving supplementary motor cortex. *Neurosurgery* 11 447–455. 10.1227/NEU.0000000000000847 26087004 PMC4540609

[B2] Amunts K. Zilles K. (2015). Architectonic mapping of the human brain beyond brodmann. *Neuron* 88 1086–1107. 10.1016/j.neuron.2015.12.001 26687219

[B3] Bailey P. (1951). *The isocortex of man.* Urbana, IL: University of Illinois Press.

[B4] Baker C. Burks J. Briggs R. Conner A. Glenn C. Sali G. (2018b). A connectomic atlas of the *human* cerebrum-chapter 1: Introduction, methods, and significance. *Oper. Neurosurg.* 15 S1–S9. 10.1093/ons/opy253 30260422 PMC6887907

[B5] Baker C. Burks J. Briggs R. Conner A. Glenn C. Morgan J. (2018a). A connectomic atlas of the human cerebrum-chapter 2: The lateral frontal lobe. *Oper. Neurosurg.* 15 S10–S74. 10.1093/ons/opy254 30260426 PMC6887966

[B6] Baker C. Burks J. Briggs R. Stafford J. Conner A. Glenn C. (2018c). A connectomic atlas of the human cerebrum-chapter 4: The medial frontal lobe, anterior cingulate gyrus, and orbitofrontal cortex. *Oper. Neurosurg.* 15 S122–S174. 10.1093/ons/opy257 30260441 PMC6887970

[B7] Bannur U. Rajshekhar V. (2000). Post operative supplementary motor area syndrome: Clinical features and outcome. *Br. J. Neurosurg.* 14 204–210. 10.1080/026886900408379 10912196

[B8] Ben Shalom D. Bonneh Y. (2019). Editorial: The medial prefrontal cortex and integration in ASD and typical cognition. *Front. Hum. Neurosci.* 13:74. 10.3389/fnhum.2019.00074 30890925 PMC6414195

[B9] Botvinick M. An J. (2009). Goal-directed decision making in prefrontal cortex: A computational framework. *Adv. Neural. Inf. Process Syst.* 21 169–176. 25258502 PMC4171955

[B10] Bressler S. Menon V. (2010). Large-scale brain networks in cognition: Emerging methods and principles. *Trends Cogn. Sci*. 14 277–290. 10.1016/j.tics.2010.04.004 20493761

[B11] Briggs R. Allan P. Poologaindran A. Dadario N. Young I. Ahsan S. (2021a). The frontal aslant tract and supplementary motor area syndrome: Moving towards a connectomic initiation axis. *Cancers* 13:1116. 10.3390/cancers13051116 33807749 PMC7961364

[B12] Briggs R. Lin Y. Dadario N. Kim S. Young I. Bai M. (2021b). Anatomy and white matter connections of the middle frontal gyrus. *World Neurosurg*. 150 e520–e529. 10.1016/j.wneu.2021.03.045 33744423

[B13] Buckner R. (2013). The brain’s default network: Origins and implications for the study of psychosis. *Dialogues Clin. Neurosci*. 15 351–358. 10.31887/DCNS.2013.15.3/rbuckner 24174906 PMC3811106

[B14] Cheng K. Fujita H. Kanno I. Miura S. Tanaka K. (1995). Human cortical regions activated by wide-field visual motion: An H2(15)O PET study. *J. Neurophysiol.* 74 413–427. 10.1152/jn.1995.74.1.413 7472342

[B15] Conner A. Briggs R. Sali G. Rahimi M. Baker C. Burks J. (2018). A connectomic atlas of the human cerebrum-chapter 13: Tractographic description of the inferior fronto-occipital fasciculus. *Oper. Neurosurg.* 15 S436–S443. 10.1093/ons/opy267 30260438 PMC6890527

[B16] Courtney S. Petit L. Haxby J. Ungerleider L. (1998). The role of prefrontal cortex in working memory: Examining the contents of consciousness. *Philos. Trans. R. Soc. B Biol. Sci*. 353 1819–1828. 10.1098/rstb.1998.0334 9854254 PMC1692423

[B17] Dadario N. Tabor J. Silverstein J. Sun X. DAmico R. (2021). Postoperative focal lower extremity supplementary motor area syndrome: Case report and review of the literature. *Neurodiagn. J.* 61 169–185. 10.1080/21646821.2021.1991716 34781833

[B18] Darby R. Joutsa J. Burke M. Fox M. (2018). Lesion network localization of free will. *Proc. Natl. Acad. Sci. U.S.A.* 115 10792–10797. 10.1073/pnas.1814117115 30275309 PMC6196503

[B19] De Carli D. Garreffa G. Colonnese C. Giulietti G. Labruna L. Briselli E. (2007). Identification of activated regions during a language task. *Magn. Reson. Imaging* 25 933–938. 10.1016/j.mri.2007.03.031 17524589

[B20] du Boisgueheneuc F. Levy R. Volle E. Seassau M. Duffau H. Kinkingnehun S. (2006). Functions of the left superior frontal gyrus in humans: A lesion study. *Brain* 129 3315–3328. 10.1093/brain/awl244 16984899

[B21] Duncan J. Owen A. (2000). Common regions of the human frontal lobe recruited by diverse cognitive demands. *Trends Neurosci*. 23 475–483. 10.1016/s0166-2236(00)01633-7 11006464

[B22] Einstein E. Dadario N. Khilji H. Silverstein J. Sughrue M. D’Amico R. (2022). Transcranial magnetic stimulation for post-operative neurorehabilitation in neuro-oncology: A review of the literature and future directions. *J. Neurooncol.* 157 435–443. 10.1007/s11060-022-03987-9 35338454

[B23] Fox P. Ingham R. Ingham J. Zamarripa F. Xiong J. Lancaster J. (2000). Brain correlates of stuttering and syllable production. A PET performance-correlation analysis. *Brain* 123 1985–2004. 10.1093/brain/123.10.1985 11004117

[B24] Freedman M. Alexander M. Naeser M. (1984). Anatomic basis of transcortical motor aphasia. *Neurology* 34 409–417. 10.1212/wnl.34.4.409 6538298

[B25] Fuster J. (2015). *The prefrontal cortex.* Cambridge, MA: Academic press.

[B26] Garey L. (1994). *Brodmann’s localisation in the cerebral cortex.* Singapore: World Scientific Publishing Company.

[B27] Glasser M. Coalson T. Robinson E. Hacker C. Harwell J. Yacoub E. (2016). A multi-modal parcellation of human cerebral cortex. *Nature* 536 171–178. 10.1038/nature18933 27437579 PMC4990127

[B28] Godefroy V. Batrancourt B. Charron S. Bouzigues A. Bendetowicz D. Carle G. (2022). Functional connectivity correlates of reduced goal-directed behaviors in behavioural variant frontotemporal dementia. *Brain Struct. Funct*. 227 2971–2989. 10.1007/s00429-022-02519-5 35751676 PMC9653340

[B29] Goldman-Rakic P. (1987). *Handbook of physiology-The nervous system.* Rockville, MD: American Physiological Society.

[B30] Hai T. Swansburg R. Kahl C. Frank H. Stone K. Lemay J. (2022). Right superior frontal gyrus cortical thickness in pediatric ADHD. *J. Atten. Disord.* 26 1895–1906. 10.1177/10870547221110918 35815438 PMC9605998

[B31] Helm K. Viol K. Weiger T. Tass P. Grefkes C. Del Monte D. (2018). Neuronal connectivity in major depressive disorder: A systematic review. *Neuropsychiatr. Dis. Treat*. 14 2715–2737. 10.2147/NDT.S170989 30425491 PMC6200438

[B32] Holmes A. Pizzagalli D. (2008). Spatiotemporal dynamics of error processing dysfunctions in major depressive disorder. *Arch. Gen. Psychiatry* 65 179–188. 10.1001/archgenpsychiatry.2007.19 18250256 PMC2587280

[B33] Hu S. Ide J. Zhang S. Li C. (2016). The right superior frontal gyrus and individual variation in proactive control of impulsive response. *J. Neurosci.* 36 12688–12696. 10.1523/JNEUROSCI.1175-16.2016 27974616 PMC5157110

[B34] Kübler A. Dixon V. Garavan H. (2006). Automaticity and reestablishment of executive control-an fMRI study. *J. Cogn. Neurosci.* 18 1331–1342. 10.1162/jocn.2006.18.8.1331 16859418

[B35] Lanzilotto M. Perciavalle V. Lucchetti C. (2013). Auditory and visual systems organization in Brodmann Area 8 for gaze-shift control: Where we do not see, we can hear. *Front. Behav. Neurosci.* 7:198. 10.3389/fnbeh.2013.00198 24339805 PMC3857530

[B36] Luria A. (2012). *Higher cortical functions in man.* Berlin: Springer Science & Business Media.

[B37] Malouin F. Richards C. Jackson P. Dumas F. Doyon J. (2003). Brain activations during motor imagery of locomotor-related tasks: A PET study. *Hum. Brain Mapp*. 19 47–62. 10.1002/hbm.10103 12731103 PMC6872050

[B38] Mandonnet E. Sarubbo S. Duffau H. (2017). Proposal of an optimized strategy for intraoperative testing of speech and language during awake mapping. *Neurosurg. Rev.* 40 29–35. 10.1007/s10143-016-0723-x 27194132

[B39] Marques R. Vieira L. Marques D. Cantilino A. (2019). Transcranial magnetic stimulation of the medial prefrontal cortex for psychiatric disorders: A systematic review. *Braz. J. Psychiatry* 41 447–457. 10.1590/1516-4446-2019-0344 31166547 PMC6796817

[B40] Martino J. Brogna C. Robles S. Vergani F. Duffau H. (2010). Anatomic dissection of the inferior fronto-occipital fasciculus revisited in the lights of brain stimulation data. *Cortex* 46 691–699. 10.1016/j.cortex.2009.07.015 19775684

[B41] Matsumura M. Sadato N. Kochiyama T. Nakamura S. Naito E. Matsunami K. (2004). Role of the cerebellum in implicit motor skill learning: A PET study. *Brain Res. Bull*. 63 471–483. 10.1016/j.brainresbull.2004.04.008 15249112

[B42] O’Neal C. Ahsan S. Dadario N. Fonseka R. Young I. Parker A. (2021). A connectivity model of the anatomic substrates underlying ideomotor apraxia: A meta-analysis of functional neuroimaging studies. *Clin. Neurol. Neurosurg*. 207:106765. 10.1016/j.clineuro.2021.106765 34237682

[B43] Petrides M. Pandya D. (2012). “Chapter 26 - The frontal cortex,” in *The human nervous system (third edition)*, eds Mai J. Paxinos G. (Berlin: Academic Press), 988–1011.

[B44] Poologaindran A. Lowe S. Sughrue M. (2020). The cortical organization of language: Distilling human connectome insights for supratentorial neurosurgery. *J. Neurosurg*. 134 1959–1966. 10.3171/2020.5.JNS191281 32736348

[B45] Poologaindran A. Profyris C. Young I. Dadario N. Ahsan S. Chendeb K. (2022). Interventional neurorehabilitation for promoting functional recovery post-craniotomy: A proof-of-concept. *Sci. Rep*. 12:3039. 10.1038/s41598-022-06766-8 35197490 PMC8866464

[B46] Postle B. Stern C. Rosen B. Corkin S. (2000). An fMRI investigation of cortical contributions to spatial and nonspatial visual working memory. *Neuroimage* 11 409–423. 10.1006/nimg.2000.0570 10806028

[B47] Price C. Green D. von Studnitz R. (1999). A functional imaging study of translation and language switching. *Brain* 122 2221–2235. 10.1093/brain/122.12.2221 10581218

[B48] Rämä P. Martinkauppi S. Linnankoski I. Koivisto J. Aronen H. Carlson S. (2001). Working memory of identification of emotional vocal expressions: An fMRI study. *Neuroimage* 13 1090–1091. 10.1006/nimg.2001.0777 11352614

[B49] Rapcsak S. Rubens A. (1994). *Localization and neuroimaging in neuropsychology.* San Diego, CA: Academic Press.

[B50] Reser D. Burman K. Yu H. Chaplin T. Richardson K. Worthy K. (2013). Contrasting patterns of cortical input to architectural subdivisions of the area 8 complex: A retrograde tracing study in marmoset monkeys. *Cereb. Cortex* 23 1901–1922. 10.1093/cercor/bhs177 22735155 PMC3698368

[B51] Rogers M. Kasai K. Koji M. Fukuda R. Iwanami A. Nakagome K. (2004). Executive and prefrontal dysfunction in unipolar depression: A review of neuropsychological and imaging evidence. *Neurosci. Res.* 50 1–11. 10.1016/j.neures.2004.05.003 15288493

[B52] Rose J. McClernon F. Froeliger B. Behm F. Preud’homme X. Krystal A. (2011). Repetitive transcranial magnetic stimulation of the superior frontal gyrus modulates craving for cigarettes. *Biol. Psychiatry* 70 794–799. 10.1016/j.biopsych.2011.05.031 21762878

[B53] Rotge J. Langbour N. Guehl D. Bioulac B. Jaafari N. Allard M. (2010). Gray matter alterations in obsessive-compulsive disorder: An anatomic likelihood estimation meta-analysis. *Neuropsychopharmacology* 35 686–691. 10.1038/npp.2009.175 19890260 PMC3055616

[B54] Ruan J. Bludau S. Palomero-Gallagher N. Caspers S. Mohlberg H. Eickhoff S. (2018). Cytoarchitecture, probability maps, and functions of the human supplementary and pre-supplementary motor areas. *Brain Struct. Funct.* 223 4169–4186. 10.1007/s00429-018-1738-6 30187192 PMC6267244

[B55] Rubens A. Whitakeer H. Whitaker H. (1976). *Studies in neurolinguistics.* Amsterdam: Elsevier.

[B56] Sarkissov S. Filimonoff I. Kononowa E. Preobraschenskaja I. Kukuew L. (1955). *Atlas of the cytoarchitectonics of the human cerebral cortex.* Moscow: Medgiz.

[B57] Shahab Q. Young I. Dadario N. Tanglay O. Nicholas P. Lin Y. (2022). A connectivity model of the anatomic substrates underlying Gerstmann syndrome. *Brain Commun.* 4:fcac140. 10.1093/braincomms/fcac140 35706977 PMC9189613

[B58] Sheets J. Briggs R. Young I. Bai M. Lin Y. Poologaindran A. (2021). Parcellation-based modeling of the supplementary motor area. *J. Neurol. Sci.* 421:117322. 10.1016/j.jns.2021.117322 33497952

[B59] Shen Y. Yuan Y. Wang M. Zhi Y. Wang J. Wang L. (2020). Dysfunction in superior frontal gyrus associated with diphasic dyskinesia in Parkinson’s disease. *NPJ Parkinsons Dis.* 6:30. 10.1038/s41531-020-00133-y 33145398 PMC7603392

[B60] Siegle G. Thompson W. Carter C. Steinhauer S. Thase M. (2007). Increased amygdala and decreased dorsolateral prefrontal BOLD responses in unipolar depression: Related and independent features. *Biol. Psychiatry* 61 198–209. 10.1016/j.biopsych.2006.05.048 17027931

[B61] Stephens T. Young I. O’Neal C. Dadario N. Briggs R. Teo C. (2021). Akinetic mutism reversed by inferior parietal lobule repetitive theta burst stimulation: Can we restore default mode network function for therapeutic benefit? *Brain Behav.* 11:e02180. 10.1002/brb3.2180 34145791 PMC8413751

[B62] Tanglay O. Young I. Dadario N. Briggs R. Fonseka R. Dhanaraj V. (2022). Anatomy and white-matter connections of the precuneus. *Brain Imaging Behav.* 16 574–586. 10.1007/s11682-021-00529-1 34448064

[B63] Usami K. Matsumoto R. Kunieda T. Shimotake A. Matsuhashi M. Miyamoto S. (2013). Pre-SMA actively engages in conflict processing in human: A combined study of epicortical ERPs and direct cortical stimulation. *Neuropsychologia* 51 1011–1017. 10.1016/j.neuropsychologia.2013.02.002 23411010

[B64] Vogt C. Vogt O. (1919). *Allgemeine ergebnisse unserer hirnforschung.* Germany: JA Barth.

[B65] Volz K. Schubotz R. von Cramon D. (2004). Why am I unsure? Internal and external attributions of uncertainty dissociated by fMRI. *Neuroimage* 21 848–857. 10.1016/j.neuroimage.2003.10.028 15006651

[B66] Volz K. Schubotz R. von Cramon D. (2005). Variants of uncertainty in decision-making and their neural correlates. *Brain Res. Bull.* 67 403–412. 10.1016/j.brainresbull.2005.06.011 16216687

[B67] von Economo C. Koskinas G. (1925). *Die cytoarchitektonik der hirnrinde des erwachsenen menschen.* Berlin: Springer.

[B68] Wu Y. Sun D. Wang Y. Wang Y. (2016). Subcomponents and connectivity of the inferior fronto-occipital fasciculus revealed by diffusion spectrum imaging fiber tracking. *Front. Neuroanat.* 10:88. 10.3389/fnana.2016.00088 27721745 PMC5033953

[B69] Yang C. Hu Y. Talishinsky A. Potter C. Calva C. Ramsey L. (2022). Medial prefrontal cortex and anteromedial thalamus interaction regulates goal-directed behavior and dopaminergic neuron activity. *Nat. Commun.* 13:1386. 10.1038/s41467-022-28892-7 35296648 PMC8927595

[B70] Yun J. Jang J. Jung W. Shin N. Kim S. Hwang J. (2017). Executive dysfunction in obsessive-compulsive disorder and anterior cingulate-based resting state functional connectivity. *Psychiatry Investig.* 14 333–343. 10.4306/pi.2017.14.3.333 28539952 PMC5440436

[B71] Zilles K. Amunts K. (2010). Centenary of Brodmann’s map–conception and fate. *Nat. Rev. Neurosci.* 11 139–145. 10.1038/nrn2776 20046193

